# Supplementary material for: The complete chloroplast genome sequence of Gentiana lawrencei var. farreri (Gentianaceae) and comparative analysis with its congeneric species
Source: PeerJ. 2016 Sep 29;4:e2540. doi: 10.7717/peerj.2540 (PMC5047142; doi:10.7717/peerj.2540)
Supplement: Table S1 [file peerj-04-2540-s001.docx]

| Clade | Regions | Sequence (5’-3’) |
| --- | --- | --- |
| For junction | Junction of NODE2-NODE5 | F: AGCATTCTTCCGCTTTCA |
|  |  | R: ACCAATTTCGCCATACCC |
|  | Junction of NODE5-NODE1(4) | F: TCTTCGCCTCTTATGTAT |
|  |  | R: TTGTCTTTTCGTCTTGTC |
|  | Junction of NODE1(4)-NODE6(5) | F: ATCCCTTTGGTGTCATTG |
|  |  | R: CCTTCTCGGTTACTTCTT |
|  | Junction of NODE6(5)-NODE4 | F: GATCCGTCTTCTTCACTT |
|  |  | R: GGTTGTATTCAGCCACTT |
|  | Junction of NODE4-NODE3 | F: AGCTTAGTTTCCCCATCA |
|  |  | R: AGAGCAGCGTGTCTACCA |
|  | Junction of NODE3-NODE6(1) | F: ATTCGGTATCAAATTCACG |
|  |  | R: TAAACAAGCACAAAGGGA |
|  | Junction of NODE6(1)-NODE1(2) | F: |
|  |  | R: |
|  | Junction of NODE1(2)-NODE2 | F: GAACCCTGTAGACCACCC |
|  |  | R: GGACGTTATTGCTCCTTT |
| For gap |  |  |
|  | GapA | F: AGCATTCTTCCGCTTTCA |
|  |  | R: ACCAATTTCGCCATACCC |
|  | GapB | F: TGGGATCACTTCTTATGG |
|  |  | R: AAACCCTCCTCTATCTGTT |
|  | GapC | F: ATTGATAAGATTTCTCCCTT |
|  |  | R: AGGCTTACTTCGCATTTA |
|  | GapD | F: CGAGTGTAATAGGAGCAT |
|  |  | R: TCCAGTTAGTAAGAGGGA |
| Known gene | *trnL(UAA)-trnF(GAA)* | F: GGTTCAAGTCCCTCTATCCCC |
|  |  | R: GGTTCAAGTCCCTCTATCCCC |
|  | *trnS(GCU)-trnG(UCC)* | F: GCCGCTTTAGTCCACTCAGC |
|  |  | R: GAACGAATCACACTTTTACCAC |
|  | *rbcL* | F: ATGTCACCACAAACAGAAAC |
|  |  | R: TCGCATGTACCTGCAGTAGC |
|  | *rpl20-rps12* | F: TTTGTTCTACGTCTCCGAGC |
|  |  | R: GTCGAGGAACATGTACTAGG |
