# Supplementary material for: The complete chloroplast genome sequence of Gentiana lawrencei var. farreri (Gentianaceae) and comparative analysis with its congeneric species
Source: PeerJ. 2016 Sep 29;4:e2540. doi: 10.7717/peerj.2540 (PMC5047142; doi:10.7717/peerj.2540)
Supplement: Table S2 [file peerj-04-2540-s002.docx]

| Taxon | Order | Family | GenBank ID |
| --- | --- | --- | --- |
| *Gentiana lawrencei*  var. farreri | Gentianales | Gentianaceae | KX096882 |
| *Gentiana straminea* | Gentianales | Gentianaceae | NC_027441 |
| *Gentiana crassicaulis* | Gentianales | Gentianaceae | NC_027442 |
| *Catharanthus roseus* | Gentianales | Apocynaceae | NC_021423 |
| *Rhazya stricta* | Gentianales | Apocynaceae | NC_024292 |
| *Nerium oleander* | Gentianales | Apocynaceae | NC_025656 |
| *Pentalinon luteum* | Gentianales | Apocynaceae | NC_025658 |
| *Oncinotis tenuiloba* | Gentianales | Apocynaceae | NC_025657 |
| *Cynanchum auriculatum* | Gentianales | Apocynaceae | NC_029460 |
| *Asclepias syriaca* | Gentianales | Apocynaceae | NC_022432 |
| *Coffea arabica* | Gentianales | Rubiaceae | NC_008535 |
| *Morinda officinalis* | Gentianales | Rubiaceae | NC_028009 |
| *Lactuca sativa* | Asterales | Asteraceae | NC_007578 |
