# Supplementary material for: The complete chloroplast genome sequence of Gentiana lawrencei var. farreri (Gentianaceae) and comparative analysis with its congeneric species
Source: PeerJ. 2016 Sep 29;4:e2540. doi: 10.7717/peerj.2540 (PMC5047142; doi:10.7717/peerj.2540)
Supplement: Table S3 [file peerj-04-2540-s003.docx]

| Category for genes | Group of gene | | | *Name of gene* |
| --- | --- | --- | --- | --- |
| Photosynthesis related genes (41 genes) | RubisCO | | | *rbcL* |
|  | photosystem Ⅰ | | | *psaA, psaB, psaC, psaI, psaJ* |
|  | Assembly/stability of photosystem Ⅰ |  |  | **ycf3, ycf4* |
|  | Photosystem Ⅱ | | | *psbA ,psbB, psbC, psbD, psbE, psbF, psbH, psbI, psbJ, psbK, psbL, psbM, psbN, psbT, psbZ* |
|  | ATP synthesis | | | *atpA, atpB, atpE, *atpF, atpH, atpI* |
|  | cytochrome b/f compelx | | | *petA, *petB, *petD, petG, petL, petN* |
|  | cytochrome c synthesis | | | *ccsA* |
|  | NADPH dehydrogenase | | | **ndhB, ndhD, ndhE, *ndhF, ndhH* |
| Transcription and translation related genes(25) | trascription | | | *rpoA, rpoB, *rpoC1, rpoC2* |
|  | ribosomal proteins | | | *rps2, rps3, rps4, rps7, rps8, rps11, *rps12, rps14, rps15, rps18, rps19,  *rpl2, rpl14, rpl16, rpl20, rpl22, rpl23, rpl32, rpl33, rpl36* |
|  | translation initiation factor |  |  | *infA* |
| RNA genes(32) | ribosomal RNA | | | *rrn16, rrn23, rrn4.5, rrn5* |
|  | transfer RNA | | | *trnA UGC, trnC GCA, trnD GUC, trnE UUC, trnF GAA, trnG GCC, trnG UCC, trnH GUG, trnI CAU, trnI GAU, trnK UUU, trnL CAA, trnL UAA, trnL UAG, trnfM-CAU, trnM CAU, trnN GUU, trnP GGG, trnP UGG, trnQ UGA, trnQ UUG, trnR ACG, trnR UCU, trnS GCU, trnS GGA, trnS UGA, trnT GGU, trnT UGU, trnV GAC, trnV UAC, trnW CCA, trnY GUA* |
| Other genes(4) | RNA processing | | | *matK* |
|  | carbon metabolism | | | *cemA* |
|  | fatty acid synthesis | | | *accD* |
|  | proteolysis | | | **clpP* |
| Genes of unknown function(4) | conserved reading frames | | | *ycf1, ycf2, ycf15, orf56* |

* Genes have intron sequences
