# Supplementary material for: The complete chloroplast genome sequence of Gentiana lawrencei var. farreri (Gentianaceae) and comparative analysis with its congeneric species
Source: PeerJ. 2016 Sep 29;4:e2540. doi: 10.7717/peerj.2540 (PMC5047142; doi:10.7717/peerj.2540)
Supplement: Table S5 [file peerj-04-2540-s005.docx]

| Unit | Length | No. | SSR start | SSR-containing region | Region | Character |
| --- | --- | --- | --- | --- | --- | --- |
| A | 12 | 1 | 6482 | IGS (trnS-GCU-trnG-GCC) | LSC | + |
|  | 11 | 3 | 29834 | IGS (trnT-GGU-psbD) | LSC | + |
|  |  |  | 72374 | IGS (petD-rpoA) | LSC | - |
|  |  |  | 106161 | ccsA | SSC | - |
|  | 10 | 6 | 18690 | rpoC1 | LSC | - |
|  |  |  | 25542 | IGS (rpoB-trnC-GCA) | LSC | + |
|  |  |  | 62933 | IGS (rps18-rpl20) | LSC | + |
|  |  |  | 103941 | ndhF | LSC | - |
|  |  |  | 104741 | rpl32 | LSC | - |
|  |  |  | 106496 | ccsA | LSC | - |
| T | 16 | 1 | 113670 | IGS (rps15-ycf1) | SSC | - |
|  | 13 | 3 | 3409 | matK | LSC | - |
|  |  |  | 58326 | IGS (petA-psbJ) | LSC | - |
|  |  |  | 110918 | IGS (rps15-ycf1) | SSC | - |
|  | 11 | 6 | 5991 | IGS (psbK-psbI) | LSC | - |
|  |  |  | 18497 | rpoC1 | LSC | = |
|  |  |  | 73216 | rpoA | LSC | - |
|  |  |  | 104453 | IGS (ndhF-rpl32) | SSC | + |
|  |  |  | 111961 | IGS (rps15-ycf1) | SSC | - |
|  |  |  | 114068 | IGS (rps15-ycf1) | SSC | - |
|  | 10 | 5 | 9502 | IGS (atpA-atpF) | LSC | - |
|  |  |  | 46192 | IGS (trnF-GAA-trnV-UAC) | LSC | = |
|  |  |  | 49275 | atpB | LSC | = |
|  |  |  | 109846 | IGS (rps15-ycf1) | SSC | - |
|  |  |  | 112033 | IGS (rps15-ycf1) | SSC | - |
| TAA | 12 | 1 | 113514 | IGS (rps15-ycf1) | SSC | - |
| TAT | 12 | 1 | 11975 | IGS (atpH-atpI) | LSC | - |
| TTG | 12 | 1 | 104173 | ndhF | SSC | = |
| TAAA | 12 | 1 | 27418 | IGS (petN-psbM) | LSC | = |
| TATG | 12 | 1 | 80131 | IGS (rpl23-trnI-CAU) | IRb | = |
| TATT | 12 | 1 | 69601 | IGS (psbH-petB) | LSC | - |
| TTCG | 12 | 1 | 36901 | psaB | LSC | - |
| CAATA | 15 | 1 | 69901 | IGS (psbH-petB) | LSC | - |
| TAAAA | 15 | 1 | 106965 | IGS (ccsA-ndhD) | SSC | - |

Note: =, identical with *G. straminea*; -, particular in *G. lawrencei* var. *farreri*; +, polymorphic with *G. straminea*.
